# Do we know enough to find an adjunctive therapy for cerebral malaria in African children?

**DOI:** 10.12688/f1000research.12401.1

**Published:** 2017-11-22

**Authors:** Brittany A. Riggle, Louis H. Miller, Susan K. Pierce

**Affiliations:** 1 Laboratory of Immunogenetics, National Institute of Allergy and Infectious Diseases, National Institutes of Health, Rockville, USA; 2 Laboratory of Malaria and Vector Research, National Institute of Allergy and Infectious Diseases, National Institutes of Health, Rockville, USA

**Keywords:** Cerebral Malaria, Plasmodium, Plasmodium falciparum

## Abstract

Cerebral malaria is the deadliest complication of malaria, a febrile infectious disease caused by *Plasmodium *parasite. Any of the five human *Plasmodium* species can cause disease, but, for unknown reasons, in approximately 2 million cases each year *P. falciparum* progresses to severe disease, ultimately resulting in half a million deaths. The majority of these deaths are in children under the age of five. Currently, there is no way to predict which child will progress to severe disease and there are no adjunctive therapies to halt the symptoms after onset. Herein, we discuss what is known about the disease mechanism of one form of severe malaria, cerebral malaria, and how we might exploit this understanding to rescue children in the throes of cerebral disease.

## Introduction

Malaria is an infectious disease caused by mosquito-borne parasites of the *Plasmodium* species, the deadliest of which, *Plasmodium falciparum* ( *Pf*), takes the lives of nearly 500,000 children each year in Africa alone ^1, 2^. For the vast majority of children, malaria is uncomplicated and resolves with time even without the highly effective anti-malaria drugs available today. However, in approximately 2% of *Pf*-infected children, for reasons we do not understand, malaria develops into a severe, life-threatening illness, and cerebral malaria (CM) is the most common form of severe disease ^3^. In malaria-endemic regions of Africa, CM occurs in young children, generally under the age of five. Although older children in these areas of high disease transmission are resistant to CM, they remain susceptible to uncomplicated symptomatic malaria into their adolescence because resistance to infection is rare, even among adults ^4^. Development of severe disease in adults is observed at a much higher frequency in southeast Asia as compared with Africa. Specifically, adult CM typically manifests in multi-organ failure and, in many ways, is a fundamentally different disease complication. As such, adult CM and its mechanisms and treatments are outside the scope of this review. The mechanisms that underlie susceptibility to CM in young children and resistance in older children are not known. The mortality for CM is high, estimated to be 15–25% despite treatment with anti-malarial drugs ^1^. Tragically, an additional 25% of children who recover from CM suffer from long-term sequelae that often leave them disabled with cognitive, hearing, and sight impairments as well as epilepsy ^3– 5^. Thus, developing an adjunctive therapy in addition to chemotherapy is a top public health and humanitarian priority.

In this brief review, we first describe key features of *Pf* infections in CM; chief among these is the parasite’s ability to sequester in tissue vasculature. We then turn to a description of our current understanding of the cellular and molecular mechanisms underlying CM and finally describe advances in identifying therapeutic targets for CM using mouse models.

## What are the key features of *Plasmodium falciparum* infections related to cerebral malaria?

Several clues for targets of adjunctive therapies for CM have come from our growing understanding of the biology of *Pf* infections. Malaria infections begin with the bite of a *Pf*-infected female mosquito that carries in her salivary glands a highly motile form of the parasite, called a sporozoite ^6^. As she probes a child’s skin to take a blood meal, she transmits a small number of sporozoites that rapidly enter the bloodstream and travel to the liver where they infect a small number of hepatocytes. This stage of the infection is asymptomatic. Over the next seven to ten days, the parasites proliferate, increasing in number over 10,000-fold, and differentiate into a form that infects red blood cells (RBCs): merozoites. The infected hepatocytes rupture, releasing a large bolus of merozoites that undergo rounds of RBC invasion, replication, and egress from the RBCs. It is in the blood stage of infection that all the clinical symptoms associated with both uncomplicated and severe malaria occur. Thus, CM is a malaria blood-stage disease. Nonetheless, the mortality from CM, even in children treated with anti-malarials that rapidly reduce blood-stage parasitemia, remains high. This suggests that there may be a tipping point in the disease progression after which elimination of the parasites, though critical, cannot save the child’s life.

A central feature of *Pf* parasite biology in the blood stage is the ability to avoid clearance of infected RBCs (iRBCs) by the spleen through sequestration in the tissue vasculature. Sequestration is mediated by the clonal expression of one of a large family of highly polymorphic genes, termed *var* genes. The *var* genes encode proteins expressed on the surface of iRBCs called *Pf* erythrocyte membrane protein 1 ( *Pf*EMP1) that bind to receptors on endothelial cell surfaces, thus allowing the iRBCs to sequester in venules ^7, 8^. There are over 60 *var* genes encoding *Pf*EMP1s in a parasite genome and many of these have multiple polymorphic forms ^9^. *Pf*EMP1s are large, multi-domain proteins that have been divided into three classes based on their upstream promoter sequences (Ups), chromosome location, and direction of transcription, namely A, B, and C ^9– 11^. Additionally, there are chimeric subsets B/A and B/C which have a UpsB promoter and a group A or C coding sequence, respectively ^10^. Recent analyses of the genomes of seven parasites defined over 600 conserved minimal *Pf*EMP1 domain building blocks ^11^. This classification is an important advance in that it appears to reflect critical biological functions of the *Pf*EMP1s and has allowed correlation of *Pf*EMP1 A and B/A with the severity of malaria ^12– 15^. *Pf*EMP1 domains bind to receptors on endothelial cell surfaces as a defense mechanism to allow sequestration in tissue and to avoid splenic clearance by the host. The binding of *Pf*EMP1s to endothelial cell surfaces may also serve to trigger cellular activity potentially harmful to the endothelium. At present, the affinity and specificity of these *Pf*EMP1-endothelial receptor interactions are incompletely understood. In fact, only a handful of receptors for *Pf*EMP1 have been identified. They include CD36, intercellular adhesion molecule 1 (ICAM-1), P-selectin, thrombospondin, platelet endothelial cell adhesion molecule 1 (PECAM1), E-selectin, vascular cell adhesion molecule 1 (VCAM1), complement receptor 1 (CR1), heparan sulfate, chondroitin sulfate A (CSA), gC1qR, and most recently endothelial protein C receptor (EPCR), none of which is selectively expressed only on the brain endothelium ^16^. Thus far, studies have demonstrated only a weak correlation between specific receptor expression and the development of severe disease ^16^.

Perhaps the most important feature of *Pf*EMP1s is that they are targets of naturally acquired protective antibody immunity ^17^. *Pf*EMP1s are expressed clonally by parasites, and a central element of clonal expression is the ability of an individual parasite to switch *var* gene expression. In the blood stage, as the infecting parasite clone expands, it is accompanied by a low level of switching to express new *var* genes. The host’s antibody response to the infecting *Pf*EMP1 clone functions to block the parasite-endothelial cell interactions, releasing the sequestered parasite for splenic clearance. The elimination of the targeted clone allows the expansion of a switched parasite clone that is not recognized by the antibodies targeting the infecting *Pf*EMP1. This process is iterated during a *Pf* infection ^11^.

In the context of CM, the critical question becomes: Are particular *Pf*EMP1s associated with CM and, if so, do they selectively bind to receptors on the brain endothelium and could such *Pf*EMP1-receptor interactions provide targets for development of therapeutics? There is evidence that the natural acquisition of antibody immunity to *Pf*EMP1 domains is ordered with children in endemic areas first acquiring immunity to a particular domain in group A (and B/A) *Pf*EMP1s ^10, 18^. This is an intriguing observation, suggesting that susceptibility of young children to CM is the result of infection with group A *Pf*EMP1s. Indeed, recent findings provided evidence that CM is associated with the expression of group A *Pf*EMP1 subtypes containing domain cassette 13 (DC13) and the expression of group B/A *Pf*EMP1 subtypes containing DC8 ^19^. Subsequently, receptors for DC8 and DC13 were discovered, including EPCR, a host receptor involved in endothelial cyto-protective pathways; gC1qR, a membrane protein with affinity for diverse ligands, including the globular “heads” of C1q; and ICAM-1, an endothelial cell surface glycoprotein ^20– 22^. Although these receptors are not selectively expressed in the brain, this is an exciting finding that suggests the possibility of targeting the binding of *Pf*EMP1s to these receptors for adjunctive therapy in CM.

## What do we know about the causes of cerebral malaria in children and is it sufficient to provide clues for targets for adjunctive therapy?

According to the World Health Organization (WHO), the term “cerebral malaria” describes a syndrome consisting of unarousable coma not attributable to convulsions, hypoglycemia, trauma, or other coma-causing infections, such as meningitis, in a patient with *Pf* parasitemia ^3^. However, the application of this definition is not straightforward in children in malaria-endemic Africa, and this is due in part to the difficulty in discerning whether a *Pf* peripheral parasitemia is the cause of loss of consciousness or is simply incidental in children with an unrelated cause of coma. In general, in malaria-endemic Africa, children are not taken to regional clinics until they show signs of CM, such as coma and seizures. Children who meet the definition of CM are immediately treated with anti-malarial drugs. Recovery or death usually occurs within the next 24–48 hours ^3^. Current evidence indicates that children who meet the WHO definition of CM can be divided into two groups based on the presence or absence of malaria-specific retinal changes as determined by fundoscopy ^23^. In a landmark study, Taylor *et al.* showed that upon autopsy 41 out of 42 children who had retinopathy-positive (RP)-CM had *Pf* parasites sequestered in their brains and microvasculature pathology, including petechial hemorrhages and swelling. In contrast, 15 children who died of retinopathy-negative (RN)-CM showed no parasite sequestration in their brains, suggesting that *Pf* parasites may not play a role in RN-CM and that death in RN-CM may be due to other causes ^24– 26^. However, results of recent extensive clinical evaluations provided evidence that *Pf* parasites do indeed play a role in RN-CM and that RP- and RN-CM may reflect a spectrum of illness in CM and that RN-CM may be an earlier, less severe form of the same disease ^24, 27^.

Concerning the cause of death in children, in a seminal study of 168 children with RP-CM, Seydel *et al.* provided evidence that brain swelling, as measured by magnetic resonance imaging (MRI), was associated with the risk of death: 84% of children who died showed severe brain swelling in contrast to only 27% of survivors ^28^. Moreover, serial MRI scans showed evidence of decreased brain swelling in survivors who initially had swelling. The majority of patients with severely increased brain volume had decreased cerebrospinal fluid in the prepontine space and posterior predominance and it was concluded that the most likely cause of death was brain stem herniation ^28^.

Although several possible mechanisms have been proposed to account for the pathology of CM in children, none has been established. Moreover, we do not yet know how similar the underlying mechanisms are that result in RN-CM versus RP-CM. Sequestration of iRBCs in the brain vasculature, as observed in RP-CM, could mechanically obstruct venules and is likely an important contribution to CM. In addition to mechanical obstruction, binding of iRBCs to endothelial receptors could have wide-ranging repercussions, including triggering pathological adhesion, coagulation, and inflammation; disrupting the integrity of the blood-brain barrier (BBB); and impairing vasoregulation. Clinical malaria has also been shown to be accompanied by decreases in nitric oxide, essential for endothelial homeostasis, but adjunctive nitric oxide supplementation by inhalation showed no efficacy ^29^. Thus, therapies based on disruption of iRBC surface *Pf*EMP1s and their brain endothelial receptors are of particular interest (see above), as are treatments that can modulate or reverse endothelial activation and damage, coagulation, and hemorrhaging. For example, coagulation is induced by activated thrombin, and heparin, which activates anti-thrombin III, and has been tested in severe malaria but had no effect on the outcome ^30^. Activated protein C, an anticoagulant that is diminished in severe malaria, has been administered as a therapy but has not been tested in controlled studies ^30^. Additional treatments have targeted the symptoms resulting from CM, namely brain swelling and mechanical obstruction by iRBCs with osmotic diuretics (mannitol) or phosphodiesterase inhibition (pentoxifylline), but have had limited to no success ^31, 32^. Trials involving neuro-protective drugs such as erythropoietin, anti-tumor necrosis factor, and iron chelation were equally disappointing ^33– 35^. Indeed, of over 17 clinical trials examining 11 therapies thus far, none has conclusively proven to be efficacious in the treatment of pediatric CM ^36^.

## How do we search for potential adjunctive therapies given that it is not possible to study cerebral malaria in children?

Because of the relatively rare nature of severe disease, involving only approximately 2% of malaria cases, and the limitations of studies in sick children, CM is a difficult pathology to investigate. This leaves two alternatives, namely studies *in vitro* and in animal models. Currently, there are several promising studies *in vitro* targeting brain endothelial activation ^37^ with pantethine ^38^, curcumin ^39^, and diannexin ^40^. These experimental data have the advantage of using human brain endothelial cells and human parasites but have the shortcomings of any *in vitro* system.

We would argue that the existing mouse model of CM, termed experimental CM (ECM), provides an excellent opportunity for discovery of adjunctive therapies for CM. Infection of susceptible mouse strains, including C57Bl/6, with *Plasmodium berghei* ANKA ( *Pb*A) results in a fatal neurological disease, the primary symptoms of which mirror those observed in children. However, the use of mouse models is controversial ^36, 41^. Historically, the primary objection to the use of the ECM mouse model for discovery of therapeutic targets for CM was that the model failed to replicate key pathologic features of CM in children ^36^. For example, one important drawback of the ECM model is the lack of *Pf*EMP1 orthologues in *Pb*A, precluding the study of therapeutics targeting *Pf*EMP1 ** and their receptor binding interactions. However, a recent study provided evidence that the machinery necessary for the transport of *Pf*EMP1 to the iRBC surface is conserved in *Pb*A parasites and that *Pb*A parasites deficient in components of this machinery fail to cause ECM. Thus, the mouse model may provide an opportunity to study the link between sequestration and virulence in *Pb*A-infected mice ^42^. Moreover, the current literature suggests that CM and ECM share several underlying mechanistic similarities that could prove to be desirable targets for adjunctive therapy ^41, 43^. With the use of the powerful tools available in mice, namely genetically modified mice, two-photon intravital microscopy of the brain, and genetically modified parasites, it has been established that key elements in ECM include the presence of parasites and infiltration of leukocytes in the brains of *Pb*A-infected mice ^44, 45^. Although some studies have pointed to the role of macrophages ^46^, monocytes ^47^, neutrophils ^48^, CD4 ^+^ T cells ^49^, and natural killer cells ^50^ in ECM, others have shown that neither genetic nor antibody-mediated depletion of these cells mitigated disease ^51– 54^. However, the evidence for the role of CD8 ^+^ T cells is well established, as demonstrated in a multitude of depletion and effector function assays ^44, 45, 49, 51, 52^. CD8 ^+^ parasite-specific T cells interact with the endothelium in the brains of infected mice, resulting in inflammation, brain endothelial cell activation, BBB breakdown, brain swelling, and microhemorrhages ^55^. A current model for the mechanisms underlying ECM is one in which iRBCs bind to brain endothelia which both activates the endothelium, compromising tight junction function, and allows cross-presentation of parasite antigens on major histocompatibility (MHC) class I molecules on the endothelium. Parasite-specific CD8 ^+^ T cells that are primed in the spleen are recruited to the brain, where they cause local damage to the endothelium through perforin-dependent mechanisms. The integrity of the BBB is lost and the brain swells, causing herniation of the brain stem, resulting in death. One key feature of ECM is the infiltration of CD8 ^+^ T cells into the brain of infected mice. At present, there is little evidence for or against the presence of CD8 ^+^ T cells in the brains of children with CM, an important gap in our knowledge that may be filled by modern tools such as multiplex immunohistochemistry of autopsied samples from children who died of CM.

As children are not taken for medical care until neurological signs of the disease occur, an effective therapy for CM would both halt the progression of the disease and reverse brain damage. In the mouse ECM model, the disease progresses rapidly, resulting in clinical signs of neurological damage five days post-infection (d5 p.i.) in the morning. The disease progresses through the day such that on the evening of d5 p.i. the mice have lost BBB integrity and show brain swelling and brain hemorrhages. Mice begin to die on d6 p.i. and by d7 p.i. nearly all mice succumb. A large number of therapies (more than 48) have been tested in the mouse ECM model and proved to be highly effective at blocking the development of ECM but only when administered prior to the appearance of clinical signs, generally before d4 p.i. ^36^. For example, we ^56^ and Mejia *et al.* ^57^ provided evidence that rapamycin, an inhibitor of the mammalian target of rapamycin (mTOR) that plays a central role in regulating T-cell metabolism, blocked the development of ECM when given on d4 p.i., but had little or no effect when give on d5 p.i.. In collaboration with Jonathan Powell and Barbara Slusher at the Johns Hopkins University School of Medicine, we tested a second inhibitor of activated T-cell metabolism, 6-diazo-5-oxo-L-norleucine (DON), a glutamine analog that blocks the glutaminase-mediated conversion of glutamine to glutamate. Remarkably, treatment with DON as late as d6 p.i. rescued 50% of mice from ECM and allowed brain recovery ^58^. When compared with untreated control mice that died showing brain swelling and BBB dysfunction, DON-treated mice survived and on necropsy their BBBs were functional and brain swelling had decreased. The number of T cells in brains of DON-treated mice did not change but the T cells were inactive. The rapidity with which DON acted suggests to us that its therapeutic efficacy may be due to an effect on balance of glutamine and glutamate in the brain, a possible mechanism we are actively pursuing. A second potential adjunctive target came from collaborative studies with Dorian McGavern and Phil Swanson at the National Institutes of Health. Early work demonstrated the efficacy of treatment of *Pb*A-infected CBA/Ca mice with LFA-1–specific antibodies (anti-LFA-1) ^59, 60^. Building on this previous work, we provided evidence that treatment of C57BL/6 mice with a combination of monoclonal antibodies specific for LFA-1 and VLA-4 (anti-LFA-1/VLA-4), which disrupted the integrin-dependent association of T cells with the brain endothelium, was effective at rescuing 100% of mice from ECM when administered on d5.5 p.i. and again on d6.5 p.i. ^55^. This is a promising finding as anti-LFA-1/VLA-4 (also known as efaluzimab and natalizumab, respectively) treatment is approved for use in humans ^61– 63^. To our knowledge, the only studies published to date that demonstrate treatment efficacy after the onset ECM symptoms are those using DON or anti-LFA-1/VLA-4.

## Conclusions

*Pf* CM is a deadly disease that takes the lives of hundreds of thousands of African children each year and leaves survivors with lifelong disabilities. At present, the mechanisms that underlie the brain pathology in CM are not verified, although the sequestration of *Pf* parasites in the brains of children with CM points toward possible targets for therapy. Because CM cannot be readily studied in children, the mouse ECM model is an essential tool for the identification of targets for CM therapies. Recent successes in identifying therapeutics in mice that are effective even after ECM neurological signs appear are encouraging. Our hope is that with an increasing understanding of the mechanisms that underlie ECM and their possible similarities to events in CM, we may learn just enough to find an adjunctive therapy for CM in African children.

## Abbreviations

BBB, blood-brain barrier; CM, cerebral malaria; d, day; DC, domain cassette; DON, 6-diazo-5-oxo-L-norleucine; ECM, experimental cerebral malaria; EPCR, endothelial protein C receptor; ICAM-1, intercellular adhesion molecule 1; iRBC, infected red blood cell; LFA-1, lymphocyte function-associated antigen 1; MRI, magnetic resonance imaging; *Pb*A, *Plasmodium berghei* ANKA; *Pf*, *Plasmodium falciparum*; *Pf*EMP1, *Plasmodium falciparum* erythrocyte membrane protein 1; p.i., post infection; RBC, red blood cell; RN, retinopathy-negative; RN-CM, retinopathy-negative cerebral malaria; RP, retinopathy-positive; RP-CM, retinopathy-positive cerebral malaria; Ups, upstream promoter sequences; WHO, World Health Organization.
